# Non-canonical Mechanisms of Presynaptic Kainate Receptors Controlling Glutamate Release

**DOI:** 10.3389/fnmol.2018.00128

**Published:** 2018-04-20

**Authors:** José V. Negrete-Díaz, Talvinder S. Sihra, Gonzalo Flores, Antonio Rodríguez-Moreno

**Affiliations:** ^1^Laboratory of Cellular Neuroscience and Plasticity, Department of Physiology, Anatomy and Cell Biology, University Pablo de Olavide, Seville, Spain; ^2^División de Ciencias de la Salud e Ingenierías, Universidad de Guanajuato, Guanajuato, Mexico; ^3^Department of Neuroscience, Physiology and Pharmacology, University College London, London, United Kingdom; ^4^Laboratorio de Neuropsiquiatría, Instituto de Fisiología, Benemérita Universidad Autónoma de Puebla, Puebla, Mexico

**Keywords:** metabotropic, kainate receptors, presynaptic, glutamate, G protein, protein kinase A, protein kinase C

## Abstract

A metabotropic modus operandi for kainate receptors (KARs) was first discovered in 1998 modulating GABA release. These receptors have been also found to modulate glutamate release at different synapses in several brain regions. Mechanistically, a general biphasic mechanism for modulating glutamate release by presynaptic KARs with metabotropic actions has emerged, with low KA concentrations invoking an increase in glutamate release, whereas higher concentrations of KA mediate a decrease in the release of this neurotransmitter. The molecular mechanisms underpinning the opposite modulation of glutamate release are distinct, with a G-protein-independent, adenylate cyclase (AC)- and protein kinase A (PKA)-dependent mechanism mediating the facilitation of glutamate release, while a G-protein dependent mechanism (with or without protein kinase recruitment) is involved in the decrease of neurotransmitter release. In the present review, we revisit the mechanisms underlying the non-canonical modus operandi of KARs effecting the bimodal control of glutamatergic transmission in different brain regions, and address the possible functions that this modulation may support.

## Introduction

Kainate receptors (KARs) together with AMPA and NMDA receptors have been classically grouped in the ionotropic family of glutamate receptors distinguishing them from the heptahelical metabotropic glutamate receptors family with canonical G-protein coupling. This classification based on structure is, however, becoming more and more complex from a functional viewpoint, given that kainate, AMPA and NMDA receptors, are all proposed as also having metabotropic and non-canonical actions (reviewed in Valbuena and Lerma, 2016; Bouvier et al., 2018). Thus the KAR, while structurally an “ionotropic” glutamate receptor, has espoused metabotropic and non-canonical actions through now extensive studies. Beginning in 1998, when the first metabotropic physiological action was described in the KAR modulation of inhibitory GABA release in the hippocampus (Rodríguez-Moreno and Lerma, 1998), these receptors have since been shown to have metabotropic/non-canonical actions modulating excitatory neurotransmitter release, at a number of glutamatergic synapses. Here, we use the term “metabotropic” when G protein involvement has been demonstrated, and “non-canonical” when no G-protein necessity has been found, but the mechanism nevertheless involves the activation of the adenylate cyclase/cAMP/protein kinase A (AC/cAMP/PKA) intracellular signaling cascade, that is patently metabotropic, but the difference being that the AC is activated by Ca^2+^-calmodulin and not by a Gs protein.

Typically, KARs mediate synaptic transmission postsynaptically, and presynaptically modulate neurotransmitter release at several synapses (for reviews, see Lerma and Marques, 2013; Sihra and Rodríguez-Moreno, 2013). Additionally, KARs contribute to short- and long-term plasticity (for review, see Sihra et al., 2014). In their modulatory role, KARs fine control both GABA and glutamate release (for review Rodríguez-Moreno and Sihra, 2007a,b; Lerma and Marques, 2013; Sihra and Rodríguez-Moreno, 2013). Although early work on this metabotropic role of presynaptic KARs evinced an inhibitory action on neurotransmitter release, subsequent studies revealed a biphasic modulation, whereby at high agonist concentrations, KARs effected release depression, while relatively “low” agonist concentrations mediated release facilitation. Originally, it was ground-breaking work with hippocampal GABAergic synapses that espoused a KAR-mediated suppression of GABA release (Clarke et al., 1997; Rodríguez-Moreno et al., 1997), but subsequent investigations revealed that a facilitation of GABA release mediated by KARs could also be observed (Cossart et al., 2001). Similarly, biphasic modulation is also evident in the modulation of glutamate release by KARs. Thus, KARs invoke both inhibitory effects (Vignes et al., 1998; Contractor et al., 2000, 2003; Kamiya and Ozawa, 2000; Schmitz et al., 2000; Negrete-Díaz et al., 2006, 2007, 2012; Lyon et al., 2011), and facilitatory modulation of glutamate release (Bortolotto et al., 1999; Contractor et al., 2001, 2003; Lauri et al., 2001a,b; Breustedt and Schmitz, 2004; Rodríguez-Moreno and Sihra, 2004, 2013; Pinheiro et al., 2007; Scott et al., 2008; Fernandes et al., 2009; Andrade-Talavera et al., 2012, 2013).

Here we describe the known *non-canonical* actions of KARs involved in the facilitatory and inhibitory modulation of glutamate release at different synapses in defined brain regions, and their possible physiological function therein. In the working definition of *non-canonical*, we include all actions of KARs, outwith their membrane depolarizing activity, but including those that invoke activation of intracellular signaling pathways. In the latter mode, we consider: (i) directly metabotropic actions, i.e., those demonstrably involving G-proteins; and (ii) non-canonical actions that operate through activation of intracellular cascades, but independently of G protein involvement, i.e., those mediated through rises in intracellular Ca^2+^ as the second messenger. We further discuss the downstream protein kinases recruited in the mediation of this metabotropic regulation of glutamate release by KARs.

## Hippocampus

### Non-canonical Control of Glutamate Release by KAR Activation at CA3-CA1 Synapses

#### Depression of Glutamate Release

In the hippocampus, Frerking et al. (2001) first reported a direct metabotropic action for KARs in the depression of glutamatergic transmission at CA3-CA1 synapses. The presynaptic localization of this KAR-mediated modulation was confirmed based on fluctuation analysis and paired-pulse analysis. The metabotropic mechanistic assignment stemmed from the modulation requiring G-protein activation, such that the KAR-mediated depression of the EPSP was abrogated in the presence of G-protein inhibitors N-ethylmaleimide (NEM) and pertussis toxin (PTX). Notably, judging by the lack of effect of the broad spectrum protein kinase inhibitor H-7, this regulation did not require a diffusible second messenger and protein kinase cascade. Rather, this modulation was found to be compartmentalized to the plasma membrane in that membrane-delimited beta gamma subunits of G_i/o_ directly inhibit presynaptic Ca^2+^ channels to restrain glutamate release. Additionally, this depression of glutamate release was found to converge mechanistically with presynaptic inhibition mediated by adenosine and GABA_B_ receptors as the effect of the KAR agonist ATPA depressing glutamate release was, in part, occluded by prior activation of GABA_B_ or adenosine receptors (Partovi and Frerking, 2006).

A KAR-mediated suppression of presynaptic Ca^2+^ channel activity in Schaffer collateral (SC) terminals was supported by Kamiya and Ozawa (1998), who reported that KAR activation elicited a reduction in intracellular Ca^2+^ concentration that correlated with glutamate release inhibition. This KAR-mediated decrease in Ca^2+^ channel activity was shown not to be a consequence of any reduced excitability of the nerve terminal given that the presynaptic (afferent) fiber volley was unaffected upon application of KA. Thus the modulation by KARs observed, could not be attributed to the canonical, ionotropic, depolarizing influences of KAR activation. In slices, KA application does produce an inward current in experiments showing decreased glutamate release by KAR activation (Chittajallu et al., 1996), but this inward current recovers quickly and completely, in stark contrast to the eEPSC which is long-lasting after KA treatment. Collectively, the data point to SC nerve terminals expressing autoregulatory KARs that operate in a metabotropic mode, to putatively reduce voltage-dependent Ca^2+^ channel activity, and thereby inhibit glutamate release.

Studies with the CA3-CA1 pyramidal cell (PC) synapses during development (Lauri et al., 2006; Sallert et al., 2007), have corroborated the metabotropic operation of KARs in suppressing glutamate release. Here, GluK1-containing KARs are tonically active, inhibiting glutamate release, an effect that was prevented in the presence of PTX and PKC inhibitors, thereby invoking a possible role of this function in synaptic maturation (Vesikansa et al., 2007). Together with studies on the CA3 SC input to CA1 PCs, other studies have looked at the CA3 PC association/commissural (A/C) fiber projection, for which the terminals synapse onto the apical dendrites of CA1 PC in the *stratum radiatum*. Indeed, the A/C terminals therein express KARs that suppress glutamate release. Given the common origin of projection from CA3 PCs, it would be interesting to test experimentally, the hypothesis that SC and A/C terminals share a common metabotropic mechanism of KAR-mediated inhibition of glutamate release.

At CA3 SC-CA1 synapses therefore, KAR activation consistently inhibits glutamate release and, for the moment, no facilitation of glutamate release has been described. In future studies, a possible facilitation of glutamate release and the mechanism involved should be investigated in this synapse to determine whether or not the aforementioned bimodal regulation of neurotransmitter release by KARs is a general mechanism.

#### Possible Function

Evidentially, the metabotropic action of KARs at SC-CA1 synapse is prominent during hippocampal development (Lauri et al., 2006; Sallert et al., 2007) and is developmentally regulated. Thus, in the mouse neonate hippocampus, KARs are tonically activated by glutamate and set a low probability of release depressing glutamate release via a G-protein dependent mechanism. This favors transmission during high frequency bursts of activity, typical of developing networks. With maturation, the tonic activation of these receptors is lost, causing an increase in the probability of release, allowing changes in the physiology of these synapses to favor a mature neuron mode of transmission. The same action of KARs has been described at MF-CA3 synapses, where a PTX sensitive mechanism involves the depression of glutamate release, here also requiring the activation of PKC (Lauri et al., 2005). These results indicate that KARs have a role in synaptic maturation and in the control of network oscillations during development. In this respect it is interesting that GluK1-containing KARs have been described to increase the number of synapses and frequency of excitatory miniature responses, an effect that is blocked by PKC inhibition (Vesikansa et al., 2007).

### Non-canonical Control of Glutamate Release by KAR Activation at MF-CA3 Synapses

The non-canonical actions of KARs modulating glutamate release have been extensively studied at the dentate gyrus MF-CA3 synapses of the hippocampus.

In contrast to the unimodal inhibitory regulation by KARs at SC-CA1 PC synapses, the receptors invoke bidirectional control of glutamate release at the MF-CA3 synapse. At low nanomolar concentrations, KA (<50 nM) application facilitates glutamate release (Contractor et al., 2000, 2003; Lauri et al., 2001a,b; Schmitz et al., 2001; Breustedt and Schmitz, 2004; Rodríguez-Moreno and Sihra, 2004; Andrade-Talavera et al., 2012), while at high nanomolar concentrations (>100 nM), the agonist inhibits glutamate release (Kamiya and Ozawa, 2000; Schmitz et al., 2000; Contractor et al., 2001, 2003; Negrete-Díaz et al., 2006, 2007; Andrade-Talavera et al., 2012). The KARs activated at this synapse are confirmed to be presynaptic by virtue of the effect of KA application on paired-pulse facilitation, the number of failures of eEPSC and changes in the coefficient of variation (1/CV^2^) which correlates with the change in synaptic response (Negrete-Díaz et al., 2006; Andrade-Talavera et al., 2012).

#### Depression of Glutamate Release

The examination of the KAR/glutamate release suppression profile reveals key metabotropic features (Negrete-Díaz et al., 2006).

First, high [KA] application at MF-CA3 PC synapses causes long-lasting effects with a slow recovery. Second, supporting the temporal arguments, pharmacological data corroborate the metabotropic mode of the KAR-mediated depression of glutamate release at MF-CA3 PC synapses. Thus, the G_i/o_ inhibitor PTX prevents the KAR-mediated depression of glutamate release in slices. Further to G_i/o_ participation in the KAR-mediated modulation, inhibition of PKA using H-89 (catalytic inhibitor), or Rp-Br-cAMP (cAMP competitor), abrogates the inhibitory effect of KA on the EPSC, with the inhibitors having already reduced the EPSC. The hypothesis arising from this is that high [KA]-evoked KAR activation causes a G_i/o_-mediated reduction in the AC/cAMP/PKA signaling cascade, to invoke a suppression of glutamate release (Negrete-Díaz et al., 2006). Interestingly, the glutamate release inhibition observed at the MF-CA3 PC synapse, and the GABA release inhibition shown at the interneuron-CA1 PC synapse, have a commonality in that both modulations are G_i/o_-dependent. However, downstream signaling differs at the two synapses in that, PKA *inhibition* underpins the KAR-mediated suppression of the *EPSC* at the glutamatergic synapse, while PKC *activation* (calphostin C-sensitive) underlies the KAR-mediated suppression of the *eIPSC* at the GABAergic synapse. As indicated previously, a role for a metabotropic role of KARs, sensitive to PTX, similar to that in SC-CA1 synapses, has also been described at MF-CA3 synapses during development (Lauri et al., 2005). Thus, during the first postnatal week, tonically active KARs depress glutamate release by a mechanism involving PTX-sensitive G-protein and PKC; with maturation this tonic activation is lost. Again this indicates that, both during development and in adulthood, a PTX-sensitive G-protein is involved in the depression of glutamate release. However, whereas in young animal the regulation involves PKC, in adults, modulation involves PKA and the AC/cAMP/PKA pathway. The substrates for phosphorylation by PKA that underpin the decrease in glutamate at this synapse remain to be determined in future experiments.

The question arises as to the functional role of the decrease in glutamate release at MF-CA3 synapses. Interestingly, the decreased AC/cAMP/PKA signaling linked to the diminution of glutamate release at these synapses may underpin the synaptic plasticity observed therein. Thus, it has been shown that the induction of KAR-mediated synaptic depression at the MF-CA3 can be occluded by low-frequency stimulation (LFS)-mediated long-term depression (LTD). Reciprocally, LTD can be abrogated by previous activation of KAR mediated depression (Negrete-Díaz et al., 2007; Lyon et al., 2011). LTD at MF-CA3 synapses is postulated to be mediated by type II mGluRs, which are coupled, through G_i/o_ activation, to a decrease in the AC/cAMP/PKA signaling (Kobayashi et al., 1996; Yokoi et al., 1996; Tzounopoulos et al., 1998). From this, it is evident that, in this form of LTD, the signaling from two diverse glutamate receptors, viz. KAR (in a metabotropic guise) and type II mGluRs, operationally act identically and, indeed, are mutually occlusive when applied consecutively. A similar collusion is recapitulated with long-term potentiation (LTP) at MF-CA3 PC synapses. Thus, KAR-mediated facilitation and excitatory (type I) mGluR1 activation, both operate by enhancement of glutamate release at these synapses (Schmitz et al., 2000).

#### Facilitation of Glutamate Release

That presynaptic KARs at MF-CA3 synapses could indeed mediate a facilitation of glutamate release was first shown by Schmitz et al. (2001), who found that low (50 nM) KA concentrations activating KARs at MF-CA3 synapses increased the amplitude of NMDA currents. This facilitation was contingent on glutamate released synaptically (endogenous agonist), evoked by 25 Hz MF stimulation. Further, a presynaptic locus of action for KARs was evident from the associated decrease in PPF. Subsequent studies have lent support to these initial studies. Thus, synaptic facilitation of MF-CA3 synapses mediated by presynaptic KARs is now widely recognized (Lauri et al., 2001a,b, 2003; Ji and Stäubli, 2002; Contractor et al., 2003; Breustedt and Schmitz, 2004; Rodríguez-Moreno and Sihra, 2004; Pinheiro et al., 2007; Scott et al., 2008; Fernandes et al., 2009; Andrade-Talavera et al., 2012). Contention, albeit limited, comes from report of a lack of effect of 50–100 nM KA on the EPSC amplitude at MF-CA3 synapses (Kwon and Castillo, 2008). Given that the experimental conditions for the dissenting study were very similar to those used by other laboratories, the negative observation is difficult to reconcile with the vast majority of affirmative studies.

Mechanistically, a good body of evidence now points to the KAR-mediated enhancement of glutamate release at MF-CA3 synapses being contingent on cytosolic [Ca^2+^] increases, potentially through Ca^2+^ permeable KARs (Lauri et al., 2003; Pinheiro et al., 2007; Scott et al., 2008; Andrade-Talavera et al., 2012). Notwithstanding, there have been mixed and differing reports on the issue. Indeed, some have indicated no involvement at all of Ca^2+^ in the KAR modulation (Kamiya et al., 2002), while others have actually purported that KARs effect synaptic inhibition by decreasing Ca^2+^ influx (Kamiya and Ozawa, 1998, 2000). Nonetheless, the current consensus is that KAR-mediated enhancement of glutamate release at MF-CA3 synapses, as well as the invoked short- and long-term plasticity changes, are underpinned by initial Ca^2+^ permeation via KARs, eliciting downstream Ca^2+^-induced Ca^2+^-release from internal stores (Lauri et al., 2003; Pinheiro et al., 2007; Scott et al., 2008; Andrade-Talavera et al., 2012).

Using electrophysiological and biochemical studies in slices and hippocampal nerve terminals, our groups have shown that presynaptic KAR activation at MF-CA3 synapses by low [KA], enhances glutamate release (Andrade-Talavera et al., 2012). This facilitation is abolished by the blockade of Ca^2+^ permeable KARs using the inhibitor philanthotoxin, as proposed by Lauri et al. (2003), Pinheiro et al. (2007) and Scott et al. (2008). Further, inhibition of Ca^2+^-induced Ca^2+^-release slices with ryanodine (Berridge, 1998), abrogates the enhancement by KA, corroborating the hypothesis that the Ca^2+^ signal via KARs is amplified by Ca^2+^ mobilization from intraterminal Ca^2+^ stores.

We posited some years ago (Rodríguez-Moreno and Sihra, 2004) that the facilitatory activity of presynaptic KARs (in hippocampal synaptosomes) was mediated by a downstream mechanism involving AC/cAMP/PKA signaling, as the effect of KARs activation to facilitate glutamate release was prevented in the presence of H-89 and Rp-Br-cAMP, occluded in the presence of forskolin, and the AC mediated modulation was prevented in the presence of calmidazolium (CMZ). Such a facilitatory mechanism is congruent to the inhibitory action of KARs at MF-CA3 synapses, which is also underpinned by the activation of an AC/cAMP/PKA cascade (Negrete-Díaz et al., 2006). However, there is a fundamental difference between the facilitatory and inhibitory modes of modulation. Thus, while the *facilitation* mediated by KARs is *independent* of G-protein activation, as indicated by insensitivity to G-protein inhibitors, *inhibition* by these receptors is evidently *dependent* on G-protein activation, albeit non-canonically given that KARs are not GPCRs (see Rodríguez-Moreno and Sihra, 2007a,b). This raises a longstanding quandary as to the nature of the transduction from facilitatory KAR activation to the stimulation of AC/cAMP/PKA signaling, an issue to which we have sought to attend (Andrade-Talavera et al., 2012).

Looking to the facilitation of release by KARs, we postulate that, in the hippocampus, a G-protein independent activation of AC/cAMP/PKA cascade may occur by the increase in cytosolic [Ca^2+^] produced by KAR activation, directly activating the Ca^2+^-calmodulin stimulated AC1 and/or AC8, the major Ca^2+^ stimulated ACs in the central nervous system (for reviews, see Cooper, 2003; Wang and Storm, 2003). In support of this hypothesis, we have demonstrated that the release enhancing actions of KA are abrogated by the calmodulin antagonists, W-7 and CMZ, in slices and synaptosomes (Andrade-Talavera et al., 2012).

Ca^2+^-calmodulin-mediated stimulation of ACs have been invoked to have roles in LTP and learning and memory (Cooper, 2003; Shan et al., 2008; Zhang et al., 2011). At the same time, KAR activity has been shown to be involved in LTP at MF-CA3 synapses (Bortolotto et al., 1999; Contractor et al., 2001; Lauri et al., 2001a, 2003; Schmitz et al., 2003; Breustedt and Schmitz, 2004). It is notable in this respect that, we found that treatment of slices with W-7 to suppress Ca^2+^-calmodulin, also inhibits the induction of LTP at MF-CA3 synapses (Andrade-Talavera et al., 2012). Collectively, the data point to KAR-mediated facilitation of glutamate release and the induction of LTP at MF-CA3 synapses, sharing, at least in part, a common Ca^2+^-calmodulin/AC/cAMP/PKA-dependent intracellular transduction mechanism.

#### Possible Function

What then might be the functional role of the aforementioned modulation, particularly given that that the expression of KARs at the hippocampal MF-CA3 synapses is conspicuously high (Represa et al., 1997; Darstein et al., 2003). Experimentally, it is evident that firing of granule cell with stimulus trains is needed to drive CA3 pyramidal neurons via MF terminals (Henze et al., 2002). In this context, presynaptic KARs may serve a vital role in the patterning of the responses in CA3 function and physiology. Aberrations in spike transmission at MF-CA3 PC synapses (reduction or increase), due to inapposite KAR activity, could well lead to aberrant patterns of place cell activity and delineation of spatial fields in the CA3 region. This might be predicted to affect hippocampus-dependent behavioral tasks associated with CA3 invoked working memory (Kesner, 2007).

KARs, likely via the described metabotropic and non-canonical signaling, participate intimately in key forms long-term plasticity processes, including LTP and LTD (Bortolotto et al., 1999; Schmitz et al., 2000, 2001, 2003; Contractor et al., 2001; Lauri et al., 2001a; Negrete-Díaz et al., 2007; Lyon et al., 2011; Andrade-Talavera et al., 2012). Importantly, developmentally, KARs express metabotropic activity that mediates glutamate release modulation and network activity in response to synaptic activation (Lauri et al., 2005). Thus, during postpartum hippocampal development (postnatal week one), endogenous glutamate release regulation at CA3 glutamatergic synapses seems to occur in an *action potential-independent* manner, through tonic KAR activity.

Interestingly, and related to the possible mechanistic target for the PKA pathways described for KARs modulation of glutamate release at MF-CA3 synapses, it has been demonstrated that KA mobilizes presynaptic vesicles, an effect dependent on PKA activation (Gesolmino et al., 2013). Gesolmino et al. (2013) showed that presynaptic differentiation requires assembly of scaffolding molecules and subsequent G-protein activation, and PKA dependent phosphorylation of synapsin I influences distribution of synaptic vesicles in the growth cone in response to KAR activation; these effects were prevented in the presence of the general inhibitor of Gα subunits GDP-ß-S or by PKA inhibition by H-89. In relation to the organization of neuronal compartments, additionally, KARs have been shown to be involved in the control axon growth and synaptic differentiation. Thus, in developing hippocampus, low concentrations of KA enhance motility of axonal filopodia, whereas high concentrations of the agonist do the opposite, through a PTX-sensitive mechanism (Tashiro et al., 2003). Perhaps, temporally contingent to these changes, Ca^2+^ permeable axonal KARs promote the strength of efferent connectivity by increasing the density and differentiation of functional presynaptic release sites, through mechanisms involving PKC and/or PKA (Sakha et al., 2016).

**Figure 1:**
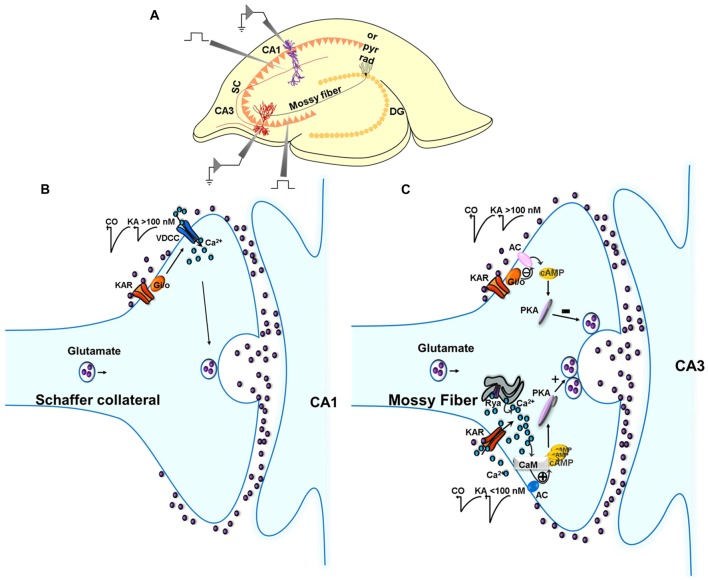
Metabotropic actions of kainate receptors (KARs) in the regulation of glutamate release in the hippocampus. **(A)** Experimental setup for studies in the hippocampus. **(B)** Metabotropic actions of KARs at CA3 Schaffer collaterals (SC)-CA1 pyramidal cell (PC) synapses. Schematic of signaling at the SC-CA1 synapse. KARs activation mediate a depression of EPSC amplitudes at SC-CA1 synapses which is abolished in slices treated with pertussis-toxin (PTX). The depression of the EPSC amplitude is not dependent on any protein kinase cascades. **(C)** Metabotropic action of KARs depressing and facilitating glutamate release at the MF-CA3 synapse. KAR activation by high concentrations of kainate (>100 nM) depresses glutamate release at MF-CA3 synapses, an effect that involves a G_i/o_ protein and the adenylate cyclase/cAMP/protein kinase A (AC/cAMP/PKA) pathway. KAR activation by low concentrations of kainate (<100 nM) facilitates glutamate release following activation of a Ca^2+^-calmodulin/AC/cAMP/PKA pathway.

In sum, in the hippocampus (Rodríguez-Moreno and Sihra, 2007a,b, Figure 1, Table 1), through a G-protein-dependent mechanism, but protein kinase-independent mechanism, KARs inhibit glutamate release at SC-CA1 synapses. KARs play a biphasic role at MF-CA3 synapses. At low [KA], KARs induce a facilitation of glutamate release mediated by G-protein-independent, Ca^2+^-calmodulin-AC/cAMP/PKA pathway. At higher [KA], KARs inhibit glutamate release through an AC/cAMP/PKA pathway with an upstream obligate input of G protein transduction, the details of which remain to be elucidated.

**Table 1:** Non-canonical actions of kainate receptors (KARs) on glutamate release.

	Low [KA]	G-protein	PKC	PKA	High [KA]	G-protein	PKC	PKA
**Hippocampus**									
SC-CA1	?	?	?	?	Depression	Yes	No	No	Frerking et al. (2001)
MF-CA3	Facilitation	No	No	Yes	Depression	Yes	No	Yes	Rodríguez-Moreno and Sihra (2004), Negrete-Díaz et al. (2006) and Andrade-Talavera et al. (2012)
**Neocortex**									
L2/3-L2/3	Facilitation	?	?	?	Depression	?	?	?	Campbell et al. (2007)
L4-L2/3
Synaptosomes	Facilitation	No	No	Yes	Facilitation	No	No	Yes	Rodríguez-Moreno and Sihra (2013)
**Thalamo-cortical synapses**	Facilitation	No	No	Yes	Depression	Yes	No	Yes	Jouhanneau et al. (2011) and Andrade-Talavera et al. (2013)
**Amygdala**									
MGN-LA	Facilitation (ATPA)	?	?	?	Depression	?	No	Yes	Shin et al. (2010) and Negrete-Díaz et al. (2012)
**Globus pallidus**	No effect				Depression	Yes	Yes	No	Jin et al. (2006)
**Spinal cord**									
DRG	Depression	?	?	?	Depression	Yes	Yes	?	Kerchner et al. (2001) and Rozas et al. (2003)

## Neocortex

### Non-canonical Facilitation of Glutamate Released Mediated by KARs Activation in the Neocortex

The actions of KARs have also been studied at cortical areas, where a facilitatory role of presynaptic KARs has been described (Perkinton and Sihra, 1999; Campbell et al., 2007; Rodríguez-Moreno and Sihra, 2013). The mechanism for this facilitation of glutamate release is the same as that found at hippocampal MF-CA3 synapses. KARs activation facilitates glutamate release and the effect requires a Ca^2+^-calmodulin/AC/CAMP/PKA pathway and does not require G-protein activation (Rodríguez-Moreno and Sihra, 2013). Looking at features of this common mechanism. This mechanism, as observed in the hippocampus (Andrade-Talavera et al., 2012), also requires an increase in cytosolic Ca^2+^, through Ca^2+^-permeable KARs and release of Ca^2+^ from intracellular stores to activate Ca^2+^-calmodulin and then the AC/cAMP/ PKA pathway as observed in synaptosomes (Rodríguez-Moreno and Sihra, 2013). As synaptosomes are devoid of functional postsynaptic elements, these experiments are demonstrative of presynaptic modulation. KAR-mediated actions are not mediated by G-proteins because modulation was unaffected by NEM treatment. Any increased cytosolic Ca^2+^ initiated by KAR activation requires a Ca^2+^-calmodulin complex to effect the modulation of glutamate release. This is postulated to be instigated by a Ca^2+^-calmodulin sensitive AC intrinsic to a AC/cAMP/PKA signaling cascade (Rodríguez-Moreno and Sihra, 2013).

## Thalamocortex

### Non-canonical Control of Glutamate Release by KAR Activation at Thalamocortical Synapses

In the thalamocortex, at synapses established between axons from ventrobasal thalamus and L4 stellate neurons from somatosensory cortex, a biphasic effect of KARs activation has been found. This KAR regulation in slices was presynaptic, because fluctuation analysis and paired-pulse changes produced the same outcomes whether measuring NMDA or/AMPA mediated postsynaptic currents. Similar to what has been found at MF-CA3 synapses, low KA concentrations mediate an enhancement of glutamate release and higher concentrations mediate a depression of glutamate release (Jouhanneau et al., 2011; Andrade-Talavera et al., 2013). Conducting studies in synaptosomes and in brain slices, common mechanistic features were established in the two preparations studied, and between synapse types. Thus, we could apply to thalamocortical synapses, previous observations in the hippocampus, where facilitation and the depression involved the AC/cAMP/PKA pathway; facilitation involving Ca^2+^-calmodulin to activate AC, and depression operating through PTX-sensitive G-protein activation (Andrade-Talavera et al., 2013). Also at thalamocortical synapses, G-protein-independent KAR-mediated facilitation of glutamate release involved an increase in cytoplasmic Ca^2+^ mediated by Ca^2+^ permeable KARs and Ca^2+^-induced Ca^2+^ release from intracellular stores, and consequent activation of AC/cAMP/PKA signaling (Andrade-Talavera et al., 2013).

#### Possible Function

Functionally, thalamocortical synapses are important for plasticity during development, and for somatosensory integration and network activity (for a review Feldman et al., 1999). KARs have been demonstrated to have a role during development as they are down-regulated during development and participate in short-term plasticity (Kidd et al., 2002).

## Amygdala

### Non-canonical Control of Glutamate Release by KAR Activation in the Amygdala

As in the hippocampus, facilitatory (Shin et al., 2010) and inhibitory (Negrete-Díaz et al., 2012) effects on glutamate release mediated by KAR activation have been described. A presynaptic AC/cAMP/PKA cascade mediating the depression of glutamate release through activation of presynaptic KARs has been shown at medial geniculate nucleus (MGN)-amygdala nucleus (LA) synapses (Negrete-Díaz et al., 2012). The effect was prevented by block of cAMP/PKA signaling by both Rp-Br-cAMP and H-89, but not by calphostin C inhibition of PKC. Whether this action is mediated by the activation of G-proteins, as in the hippocampus (Frerking et al., 2001; Negrete-Díaz et al., 2006), has not yet been determined. Relating to the facilitatory effect of KARs activation, it has not been determined yet whether this involves a metabotropic action of KARs, either via G-protein activation or activation of an AC/cAMP/PKA cascade as is becoming more and more the case in extended numbers of studies. Future studies will be needed to clarify this.

#### Possible Function

The amygdala, as a component of the limbic system, mediates emotional modulation of behavior and learning (LeDoux, 2000) and is central for the acquisition, storage and expression of conditioned fear memory. Therefore, the actions of KARs may be important for these functions.

The amygdalar KARs meditating a depression of glutamate release may have a potential protective role in mechanisms preventing network oscillations from turning to epileptogenic burst activity, as has been observed when KA concentrations are increased in the hippocampus. This depression may additionally have a utility in the suppression of seizure propagation generated by convulsive agents (Kahalilov et al., 2002; Schubert and Albrecht, 2008). KARs also have a role in plasticity at this synapse, as they are involved in LTP (Shin et al., 2010), though any role in LTD remains to be established.

KARs participate in oscillations at the theta band in the amygdala. Activation of KARs by very low concentrations of KA produces an enhancement of activity within the theta band (3–9 Hz). It will be interesting, as suggested above, to determine whether or not the effect of the activation of KARs in the amygdala involves AC/cAMP/PKA signaling as this cascade has been shown to produce a breakdown of the oscillations in the hippocampus (Fisahn et al., 2004). High agonist concentrations may be a protective mechanism preventing network oscillations to turn to epileptogenic burst activity, as observed in the hippocampus when KA concentrations are increased. Thus the depression observed in the MGN-LA amygdala synapse may have a role in oscillations and propagation of seizures and thus a neuroprotective role (Negrete-Díaz et al., 2012).

## Spinal Cord

### Non-canonical Control of Glutamate Release by KAR Activation in Spinal Cord

In spinal cord slices, the activation of KARs inhibits glutamate release and this inhibition involves a G-protein mediated mechanism (Rozas et al., 2003). In dorsal root ganglion (DRG) neurons, low KA concentrations promote neurite extension and high KAR concentrations inhibit neurite extension (Marques et al., 2013). It has been demonstrated that neurite extension requires metabotropic KARs activity and inhibition is mediated by ion channel activity. In DRG neurons, low [KA] mediated metabotropic signalling reduces CRMP2 phosphorylation to stimulate neurite elongation (Marques et al., 2013).

## Globus Pallidus

### Non-canonical Control of Glutamate Release by KAR Activation in the Globus Pallidus

In the rat globus pallidus, inhibition of glutamate release mediated by the activation of KARs with a metabotropic modus operandus involving G_i/o_ protein has been described, albeit not linked to PKA activity, but to PKC, i.e., KAR mediated modulation was prevented by treating with NEM or calphostin C, but not with H-89 (Jin et al., 2006). A facilitatory effect of KARs has not been found in the globus pallidus.

## Future Directions of Development in the Field

In the hippocampus, while synapsin I represents an established substrate for PKA in presynaptic vesicle mobilization, that might be targeted in the metabotropic modulation by KARs, additional substrates for KAR→PKA signaling require investigation. In this regard, KARs themselves are potential targets, as are a plethora of known presynaptic PKA substrates identified. Indeed, GluK2 subunits of KARs are known to be modulated by PKA, as kainate currents are potentiated by intracellular perfusion of PKA (Wang et al., 1993). Although, most of the cloned glutamate receptor subunits contain potential phosphorylation sites for PKC and Ca^2+^-calmodulin-dependent protein kinase II, only GluK2 subunit contains a consensus sequence for PKA phosphorylation (Kennelly and Krebs, 1991; Swope et al., 1992).

At thalamaocortical synapses, the question as to whether, the regulation of glutamate release by activation of presynaptic KARs is mediated by receptors located at axons or at the somatodendritic compartment needs to be resolved. Thus, it remains paramount to physically identify the subcellular location of the postulated KARs. The unequivocal delineation of KAR compartmentalization prompts direct approaches. For instance the use of immunogold-based receptor localization studies; contingent on the availability of KAR antibodies with sufficient specificity and avidity. Functional assurance of compartmentalization begs the development of reagent such as caged blockers of KARs, as has been possible for NMDA receptor localization (Rodríguez-Moreno et al., 2011; Reeve et al., 2012).

For the depressive actions of KARs on amygdalar glutamate release, the direct involvement of a G-protein has not been explored yet. Whether this depression of glutamate release mediated by the activation of KARs has a role in LTD needs to be established with more experimental work. For the facilitatory effect, as indicated above, the role of AC/cAMP/PKA pathway in the facilitation requires exploration. Additionally, whether, as in the hippocampus and the cortex, AC is activated by Ca^2+^-calmodulin following previous activation of Ca^2+^ permeable KAR and Ca^2+^-induced intracellular Ca^2+^ release, requires experimental elucidation.

In general, a paramount question remaining is whether the facilitatory and inhibitory modes of modulation of synaptic transmission by KARs involves: (i) two distinct KAR types, with different subunit compositions and possibly differential cellular localizations or; (ii) a single KAR type that mediates both modes of actions on glutamate release, but contingent on the strength of receptor activation. Another crucial issue that remains to be addressed is: how KARs (canonically ionotropic receptors) couple to G proteins when there is an obligate G-protein requirement for the KAR modulation. In this regard some advance has been made. Ruiz et al. (2005) identified a biochemical interaction between GluK5 and Gαq and Rutkowska-Wlodarczyk et al. (2015) suggesting an interaction of GluK1 ionotropic KAR subunits with Go proteins by proteomic analysis. Further, it remains to be elucidated what the precise roles of the inhibitory and facilitatory actions of KARs are in network oscillations and behavior involving the different brain regions where these modulation modes have been found. The roles of the metabotropic activities of KARs in epilepsy and excitotoxicity also require expounding. Knowing that the inhibitory and facilitatory modulatory actions of KARs on glutamate release are contingent on the activation of PKC or PKA respectively, identification and characterization of the target proteins being phosphorylated by these protein kinases, requires elucidation to ascertain the specific distal mechanisms underpinning the observed modulation by KARs. In all of the brain regions reviewed here, the subcellular compartmentalization, axonal or somatodendritic, of the KARs mediating metabotropic modulation of glutamate release is of fundamental import and requires address.

In summary, KARs may modulate glutamate release bimodally; viz. a depression or a facilitation of glutamate release. The depression of glutamate release in the known cases is mediated by G-protein activation, with one case of a protein kinase-independent (membrane-delimited) mechanism, but all other reports recording the involvement of a protein kinase activity. This protein kinase activated by KAR signaling is PKC in some studies, but alternatively, an emerging number of studies invoke the involvement of PKA activity. This PKA activation stems from an AC/cAMP/PKA pathway. KARs with a metabotropic depressive action are clearly involved in maturation during development and in the trafficking of vesicles, having a possible role in LTD and providing a protective role in preventing oscillations leading epileptic seizures. At low [KA], KARs mediate an increase in glutamate release that does not require G protein activity, but reflects non-canonical signaling, in most cases a cascade involving the Ca^2+^-calmodulin/AC/cAMP/PKA pathway. These KARs may have functions during synaptic maturation, and in LTP and other plasticity.

## Author Contributions

JVN-D, TSS, GF and AR-M contributed equally to the design and writing of the review. JVN-D made the Figure 1 and Table 1.

## Conflict of Interest Statement

The authors declare that the research was conducted in the absence of any commercial or financial relationships that could be construed as a potential conflict of interest.
